# L-arginine role for stone lower ureter: A randomized controlled trial

**DOI:** 10.1007/s00240-025-01768-3

**Published:** 2025-06-11

**Authors:** Ahmed Mahmoud Mohammed Ahmed Elsherief, Ahmed Mahmoud Riyad, Emad Abdellah Ali, Amr Alam-Eldin Ahmed, Hassan Ali Gad, Ahmed Mamdouh Abd elhameid

**Affiliations:** 1 Sohag University, Sohag, Egypt; 2 Aswan University, Aswān, Egypt

## Abstract

This study aims to evaluate the effects of L-arginine 1000 mg once daily as a medical expulsive therapy (MET) for lower ureteral stones. This prospective, randomized controlled study was conducted on 162 patients with ureteral stones; 9 patients were excluded due to loss to follow-up, and the remaining 153 patients were divided into three groups. Group I (control) included 51 patients who received a placebo. Group II included 51 patients who received 1000 mg of L-arginine once daily. Group III included 51 patients who received tamsulosin 0.4 mg once daily. There was a highly statistically significant difference between the studied groups regarding ultrasound (U/S) and plain urinary tract (PUT) findings after 4 weeks. Spontaneous stone expulsion rates (SER) in the control, L-arginine, and tamsulosin groups were 6 (11.8%), 48 (94.1%), and 16 (31.4%), respectively (*p* < 0.001). The mean ± SD of stone expulsion time in the control, L-arginine, and tamsulosin groups was 19.6 ± 5.85, 19.02 ± 5, and 20.58 ± 5.78 days, respectively (*p* < 0.001). There was no statistically significant difference between the groups regarding the number of daily colic episodes and total analgesic dosage required. However, a statistically significant difference was noted regarding stone density and hydronephrosis. It is concluded that L-arginine is more effective than tamsulosin in increasing the SER and reducing stone expulsion time, with better pain control, making it a safe and effective MET for ureteral stones.

## Introduction

Urolithiasis is a prevalent condition globally, and its incidence is rising [1]. Urinary stones are often located in the ureter, with the majority found in the distal portion. Stones smaller than 3 mm in diameter are generally more likely to pass spontaneously, while stones larger than 6 mm are less likely to do so [2].

L-arginine-derived nitric oxide (NO) is the primary inhibitory non-adrenergic, non-cholinergic (NANC) neurotransmitter in the lower urinary tract [3]. Neuronal nitric oxide synthase (nNOS)-positive axons and nerve-ending-like structures have been observed in the muscular layers of the human ureter, suggesting that ureteral relaxation may involve the NO pathway. Additionally, NOS-reactive nerves have been identified immunohistochemically in the human intravesical ureter [4]. A recent study suggests that the L-arginine/NO/cyclic GMP pathway may regulate valve function at the ureterovesical junction (UVJ) [5].

Renal colic (RC), due to urinary stone disease, affects 1–10% of individuals at some point in their lives and accounts for 2% of all emergency department admissions [6]. When a stone enters the ureter, it increases intraluminal pressure, leading to ureteric smooth muscle contractions. This pressure causes irritation and obstruction, resulting in painful spasms that are often intolerable [7].

L-arginine, a precursor of NO, plays a role in the pathophysiology of renal colic. nNOS is present in axons and neuronal endings within the human ureter, indicating a potential link between NO production and renal colic. The hypothesis is that NO may facilitate stone progression by promoting ureteral smooth muscle relaxation. However, the role of endothelial nitric oxide synthase (eNOS)—another isoenzyme involved in NO synthesis that functions in smooth muscle organs like the ureter—remains underexplored. eNOS, encoded by a gene on the long arm of chromosome 7, contributes to NO production that relaxes smooth muscles in the gastrointestinal and cardiovascular systems and influences various physiological processes [8].

This study aimed to evaluate the effects of once-daily L-arginine as a medical expulsive therapy for lower ureteral stones through a randomized controlled design.

## Patients and methods

This prospective, non-blinded, randomized controlled study was conducted on 162 patients with ureteral stones. Nine patients were excluded from the study due to loss to follow-up, and the remaining 153 patients were divided into three groups: Group I included 51 patients who received a placebo; Group II included 51 patients who received L-arginine 1000 mg once daily; and Group III included 51 patients who received tamsulosin 0.4 mg once daily.

Outcomes:

- Primary outcome: To evaluate the efficacy of L-arginine 1000 mg once daily as a MET, compared with tamsulosin and placebo, in promoting stone expulsion in patients with lower ureteral stones, measured by the SER.
- Secondary outcomes: Reduction in analgesic requirement, shorter stone expulsion time, and improvement in quality of life.

Randomization was performed using computer-generated random tables, utilizing stratified blocked randomization in a 1:1:1 ratio.

Inclusion criteria: Patients older than 18 years with distal ureteral stones between 4 and 10 mm in size, with a unilateral single stone, symptoms lasting on average for two days, no severe hydronephrosis, normal renal function, non-pregnant women, no sepsis, and those who adhered to follow-up.

Exclusion criteria: Acute azotemia, stones larger than 10 mm, solitary kidney, symptomatic urinary tract infection, impacted stones, severe hydronephrosis, multiple or bilateral stones, pregnancy, anatomical abnormalities of the urinary system, and benign prostatic hyperplasia.

### Sample size calculation and statistical analysis

The sample size was calculated according to Charan and Biswas [9], based on the primary outcome (SER) among the three study groups. Assuming SERs of 60% in the L-arginine group, 50% in the tamsulosin group, and 30% in the control group, with a significance level (alpha) of 0.05 and a statistical power of 80%, the minimum required sample size was 45 patients per group (using the Chi-square test). To account for potential dropouts, the sample size was increased to 51 patients per group, for a total of 153 patients.

Continuous data were summarized using the mean ± standard deviation (SD) for normally distributed data, and median (range) for skewed data. Categorical variables were presented as frequencies and percentages and compared using Fisher’s exact test. Statistical analysis was conducted using the Statistical Package for the Social Sciences (SPSS), version 13.0 for Windows (SPSS Inc., Chicago, IL). A p-value ≤ 0.05 was considered statistically significant. Comparisons were performed using the independent t-test for normally distributed data and the Mann–Whitney U test for non-normally distributed data.

For the secondary outcome of stone expulsion time, a standard deviation of approximately 6 days was assumed, based on previously published data. This provided sufficient power to detect clinically meaningful differences in expulsion time using one-way ANOVA.

## Methods

All patients underwent detailed history taking, physical examination, urinalysis and urine culture, renal function tests, U/S, PUT imaging, and computed tomography (CT) of the urinary tract when needed.

## Interventions

Lower ureteral stones refer to those in the distal third of the ureter, specifically the segment between the pelvic brim and the UVJ, where the ureter enters the bladder, and were diagnosed using PUT and abdominal U/S. A CT scan was performed in suspected cases. Diclofenac (100 mg per day) was used as an analgesic for episodes of renal colic. It is acknowledged that participants, knowing their treatment assignment, might report less pain, and physicians, likewise, might adjust analgesic prescribing behavior.

## Hydronephrosis grading criteria

In this study, hydronephrosis was graded using the Society for Fetal Urology (SFU) grading system as follows:

- **No hydronephrosis**: Normal renal pelvis and calyces with no signs of dilatation.
- **Splitting of calyces**: Early sign of obstruction, showing initial separation or mild widening of the calyces without full pelvic dilatation.
- **Mild hydronephrosis**: Slight separation of the pelvicalyceal system (renal pelvis) without calyceal dilatation.
- **Moderate hydronephrosis**: Dilatation of both renal pelvis and calyces with early thinning of renal parenchyma.
- **Marked (severe) hydronephrosis**: Gross dilatation of the pelvicalyceal system with significant cortical thinning, suggestive of chronic obstruction.

## Follow-Up

Patients were followed weekly for 4 weeks using U/S and/or PUT, with attention to:

- Spontaneous SER.
- Stone expulsion time (reported by the patient or confirmed radiologically).
- Number of daily colic episodes.
- Total required analgesic dosage.
- Medication side effects.

Successful outcomes were defined as complete stone passage. Stone expulsion time was defined as the number of days from treatment initiation to confirmed stone passage, either radiologically or self-reported. For patients who did not pass their stone during follow-up, a fixed value of 28 days was assigned to enable inclusion in the mean expulsion time analysis.

Criteria for treatment discontinuation or intervention included uncontrollable pain, fever, worsening hydronephrosis, failure to pass the stone after 4 weeks, urinary infection, or patient request for interventional stone removal.

A multivariate logistic regression analysis was used to identify independent predictors of spontaneous stone expulsion. The model included treatment group (L-arginine, tamsulosin, control), stone density, and degree of hydronephrosis—variables that showed baseline differences and are known to affect stone passage. Stone size and laterality were also included as covariates.

### Ethical considerations

The study was approved by the local ethics committee. All participants were informed about the study and provided written informed consent before enrollment.

## Results

A total of 153 patients with ureteral stones were divided into three groups to complete the study.

There was no statistically significant difference between the studied groups regarding age, sex, medical history, physical examination, operative history, urine culture, serum creatinine, U/S, CT urography (CTU), and number of stones detected by CTU. However, there was a highly statistically significant difference in urine analysis and PUT findings (Table 1).

As regards the distribution of stone characteristics between the studied groups, the stone was located in the lower ureter in all cases, except for one case in the L-arginine group, where the stone was in the middle ureter (p-value = 0.37; not statistically significant). The mean stone size was 6.7 ± 1.54 mm, 6.8 ± 1.29 mm, and 6.6 ± 1.58 mm in the control, L-arginine, and tamsulosin groups, respectively. The mean stone density was 962 ± 310.3, 802.3 ± 269.2, and 787.5 ± 261.4 HU in the control, L-arginine, and tamsulosin groups, respectively (p-value = 0.003; statistically significant).

The degree of hydronephrosis was variable, with absence in 18 patients: 10 (19.6%), 3 (5.9%), 5 (9.8%); splitting of calyces in 13 (25.5%), 6 (11.8%), 3 (5.9%); mild hydronephrosis in 11 (21.6%), 28 (54.9%), 23 (45.1%); moderate hydronephrosis in 17 (33.3%), 14 (27.4%), 20 (39.2%) in the control, l-arginine, and tamsulosin groups respectively, with a p-value of 0.003, which was statistically significant.

Follow-up during the 1 st and 2nd weeks showed no significant difference in U/S findings; however, there was a highly significant difference in PUT (Table 2).

In the 3rd and 4 th weeks, there was a statistically significant difference in U/S findings. PUT findings were not significant in week 3 but became significant after week 4 (Table 3).

There was no significant difference in the number of daily colic episodes and the total required analgesic dosage (p-values = 0.408 and 0.186, respectively). However, there was a highly statistically significant difference in spontaneous stone expulsion rates and stone expulsion time (p-value < 0.001) (Table 4). No medication side effects required discontinuation of treatment.

The mean stone expulsion time was calculated for all patients, including those who did not pass their stones, by assigning a fixed time of 28 days for non-expellers. No important side effects were recorded.

In multivariate logistic regression analysis, after adjusting for stone density, degree of hydronephrosis, and stone size, L-arginine remained a highly significant independent predictor of stone expulsion (OR = 54.7, 95% CI: 17.4–172.4, *p* < 0.001). Tamsulosin also showed a significant association compared to control (OR = 3.45, *p* = 0.017). Higher stone density was significantly associated with a lower likelihood of stone expulsion (OR = 0.998 per Housefield unit (HU), *p* = 0.004). Moderate hydronephrosis was positively associated with expulsion compared to no hydronephrosis (*p* = 0.043). The mean stone size was 6.7 ± 1.54 mm in the control group, 6.8 ± 1.29 mm in the L-arginine group, and 6.6 ± 1.58 mm in the tamsulosin group, with no statistically significant difference between groups (*p* = 0.89) (Table 5).

**Table 1:** Patients’ characteristics between the studied groups

Variables	Control group *N* = 51	L-Arginine group *N* = 51	Tamsulosin group *N* = 51	*p*-value
Age				
Mean ± SD	37.3 ± 7.5	35 ± 7.57	36.1 ± 8.7	0.34
**Sex**				
Male	34 (66.7%)	34 (66.7%)	30 (58.8%)	0.63
Female	17 (33.3%)	17 (33.3%)	21 (41.2%)	
**History**				
Stone formation	9 (17.6%)	5 (9.8%)	5 (9.8%)	0.43
Medical treatment	5 (9.8%)	2 (3.9%)	5 (9.8%)	
**Examination**				
DM/HTN	3 (5.9%)	2 (3.9%)	3 (5.9%)	0.97
Cardiac	3 (5.9%)	1 (1.9%)	1 (1.9%)	
HTN	2 (3.9%)	1 (1.9%)	2 (3.9%)	
DM	1 (1.9%)	0 (0%)	1 (1.9%)	
**Operative H/O**	9 (17.6%)	10 (19.6%)	6 (11.8%)	0.53
**Urine analysis**				
Pus or RBCs	1 (1.9%)	14 (27.4%)	2 (3.9%)	< 0.001*
**Culture**				
Bacterial growth	0 (0%)	3 (5.9%)	1 (1.9%)	0.16
**Creatinine**				
Mean ± SD	1.18 ± 0.32	1.11 ± 0.22	1.15 ± 0.25	0.41
**U/S & CTU**				
Right	15 (29.4%)	15 (29.4%)	15 (29.4%)	1
Left	36 (70.6%)	36 (70.6%)	36 (70.6%)	
**Number in CTU [1]**	51 (100%)	51 (100%)	51 (100%)	1

P value > 0.05: Not significant, P value ˂0.05 is statistically significant, and p˂0.001 is highly significant

**Table 2:** Follow-up between the studied groups during the 1 st and 2nd weeks

Follow up	Control group *N* = 51		L-Arginine group *N* = 51	Tamsulosin group *N* = 51	*p*-value
**1 st week U/S**					
No	1 (1.9%)		4 (7.8%)	1 (1.9%)	0.68
Splitting	5 (9.8%)		5 (9.8%)	5 (9.8%)	
Mild	23 (45.1%)		27 (52.9%)	23 (45.1%)	
Moderate	21 (41.2%)		15 (29.4%)	21 (41.2%)	
Marked	1 (1.9%)		0 (0%)	1 (1.9%)	
**2nd week U/S**					
No		1 (2%)	9 (17.6%)	4 (7.8%)	0.27
Splitting		4 (7.8%)	4 (7.8%)	3 (5.9%)	
Mild		19 (37.2%)	20 (39.2%)	18 (35.3%)	
Moderate		26 (51%)	18 (35.3%)	25 (49%)	
Marked		1 (2%)	0 (0%)	1 (2%)	
**PUT**					
1 st week Yes	51 (100%)		37 (72.5%)	51 (100%)	< 0.001*
2nd week Yes	50 (98%)		31 (60.8%)	46 (90.2%)	< 0.001*

P value > 0.05: Not significant, P value ˂0.05 is statistically significant, and p˂0.001 is highly significant

**Table 3:** Follow-up between the studied groups during the 3rd and 4 th weeks

Follow up	Control group *N* = 51	L-Arginine group *N* = 51	Tamsulosin group *N* = 51	*p*-value
**3rd week U/S**				
No	3 (5.9%)	24 (47.1%)	9 (17.6%)	< 0.001*
Splitting	8 (15.7%)	9 (17.6%)	7 (13.7%)	
Mild	10 (19.6%)	12 (23.5%)	5 (9.8%)	
Moderate	25 (49.01%)	6 (11.8%)	25 (49.01%)	
Marked	5 (9.8%)	0 (0%)	5 (9.8%)	
**4 th week U/S**				
No	6 (11.8%)	48 (94.1%)	17 (33.3%)	< 0.001*
Splitting	0 (0%)	0 (0%)	0 (0%)	
Mild	10 (19.6%)	0 (0%)	1 (2%)	
Moderate	27 (52.9%)	3 (5.9%)	25 (49%)	
Marked	8 (15.7%)	0 (0%)	8 (15.7%)	
**PUT**				
3rd week Yes	45 (88.2%)	6 (11.8%)	36 (70.6%)	0.02*
4 th week Yes	45 (88.2%)	2 (3.9%)	31 (60.8%)	< 0.001*

P value > 0.05: Not significant, P value ˂0.05 is statistically significant, and p˂0.001 is highly significant

**Table 4:** Outcome distribution between studied groups

Variables	Control group *N* = 51	L-Arginine group *N* = 51	Tamsulosin group *N* = 51	*p*-value
Spontaneous stone expulsion rates (SER)	6 (11.8%)	48 (94.1%)	16 (31.4%)	< 0.001
Number of daily colic episodes				
Mean ± SD	1.84 ± 0.67	2.01 ± 0.86	1.84 ± 0.67	0.408
**Stone expulsion time**				
No	46 (90.2%)	4 (7.8%)	34 (66.7%)	**< 0.001**
Yes	5 (9.8%)	47 (92.2%)	17 (33.3%)	
Mean ± SD	19.6 ± 5.85	19.02 ± 5	20.58 ± 5.78	
**Total required analgesic dosage**				
	1.94 ± 1.02	2.31 ± 1.04	2.13 ± 0.98	0.186

**Table 5:** Multivariate logistic regression analysis for predictors of stone expulsion

Variables	Odds Ratio (OR)	95% Confidence Interval	*p*-value
L-arginine (vs. control)	54.7	17.4–172.4	< 0.001
Tamsulosin (vs. control)	3.45	1.25–9.54	0.017
Stone density (per HU)	0.998	0.996–0.999	0.004
Moderate hydronephrosis (vs. none)	1.85	1.05–3.65	0.043
Stone size (per mm)	0.95	0.83–1.09	0.38

## Discussion

Distal ureteral stones are among the most symptomatic types of calculi. The spontaneous passage rate for distal ureteral stones is reported to range from 71 to 98% for stones < 5 mm, and 25–51% for stones measuring 5–10 mm [10–13]. To facilitate stone expulsion and reduce symptoms, MET—particularly with alpha-blockers, calcium channel blockers, or phosphodiesterase type 5 inhibitors (PDE5-Is)—is widely used, improving the passage of stones and reducing the need for analgesics [11, 14].

The inclusion of non-expelling patients with a fixed expulsion time of 28 days may have influenced the observed similarity in mean expulsion times across groups, despite differences in expulsion rates. This method ensures all participants are included in the time analysis, but may underestimate differences in early expulsion efficiency between groups.

NO has been shown to induce smooth muscle relaxation in both humans and a variety of animal species. Mastrangelo et al. [15] found that NO produced by the urothelium inhibits contractile responses in the proximal ureter of rats. Additionally, NOS has been identified in the nerve fibers of the porcine intravesical ureter, suggesting its role in mediating inhibitory neurotransmission [16]. In the human ureter, neuronal NOS-positive nerves have been observed, especially in the distal segment, where they appear crucial for peristalsis and motility at the ureterovesical junction [17]. Consequently, this provides a mechanistic rationale for using NO donors or precursors, such as L-arginine, in promoting ureteral stone expulsion.

Studies have shown that both endogenous and exogenous NO contribute to the relaxation of the intravesical ureter in pigs [16, 18]. The ureter receives its nerve supply from the renal plexus and the superior and inferior hypogastric plexus. The hypogastric and pelvic plexuses are well-connected synaptically. Activation of the pelvic plexus and cavernous nerves results in the initiation of an erection. When the cavernous nerve is stimulated, nitrergic nerve fibers release NO, leading to the relaxation of penile smooth muscles [19].

However, the specific type of neural stimulus affecting the ureter during sexual intercourse is not well-defined. It is hypothesized that nonadrenergic, noncholinergic (nitrergic) nerve endings may stimulate the distal ureter during sexual activity. This hypothesis is supported by the increased NO levels in the body, particularly in the penile cavernous tissue during erection. In support of this, a study by Omer Gokhan [20] found a higher stone expulsion rate in individuals who engaged in sexual intercourse compared to those in the tamsulosin and control groups after 2 weeks.

Omer Gokhan’s study also investigated the impact of engaging in at least three sexual intercourse**s** per week on stone expulsion rates. It found that the median expulsion time was significantly shorter in the sexual intercourse group compared to the other two groups (*P* = 0.0001), concluding that sexual intercourse enhances the likelihood of spontaneous stone expulsion and reduces expulsion time for distal ureteral stones. Additionally, studies on MET have shown that tamsulosin reduces episodes of renal colic and the need for analgesics [21]. Similarly, Omer Gokhan’s study indicated that the need for injections was lower in the sexual intercourse group compared to the control group (*P* = 0.001), supporting the idea that increased ureteral peristalsis and reduced basal tone of the ureter may contribute to these findings.

Our study found a significantly higher SER in the L-arginine group (94.1%) compared to the tamsulosin group (31.4%) and control group (11.8%). Radiological follow-up demonstrated significantly improved stone clearance in the L-arginine group at the third and fourth weeks, suggesting its superior efficacy. Although the mean expulsion time did not differ substantially among groups, the overall SER highlights the potential of L-arginine as an effective MET option for distal ureteric stones sized 4–10 mm.

These findings align with Erdoğan et al., who recommended a 3-week duration for MET in stones sized 5–10 mm to optimize outcomes and reduce unnecessary interventions [22]. While earlier studies recommended up to 6 weeks of MET [23], our findings suggest that effective results may be achieved within a shorter timeframe when using L-arginine.

Earlier studies have compared the effectiveness of alpha-blockers and PDE5i as MET. For instance, Falahatkar et al. found that tamsulosin had a higher, though not statistically significant, expulsion rate (72.7%) than tadalafil (63.6%) and placebo (56.8%) [24]. Tamsulosin also had a shorter mean expulsion time and analgesic use compared to tadalafil and the control. Thus, tamsulosin is more effective for distal ureteric stones, requiring less analgesia and achieving faster stone expulsion compared to tadalafil.

Other meta-analyses suggest tadalafil may outperform tamsulosin in some parameters, such as expulsion time and side effect profile (25, 26). The analysis revealed that tadalafil was more effective than tamsulosin in stone expulsion and had a shorter expulsion time. The study highlighted the safety of this combination therapy.

While numerous studies have reported that tadalafil is more effective than tamsulosin for facilitating stone expulsion [27–30], Siavash Falahatkar [29] reported that the SER was highest in the tamsulosin group, followed by the tadalafil and placebo groups, with rates of 72.7%, 63.6%, and 56.8%, respectively. However, this difference was not statistically significant (*P* = 0.294). Conversely, Al-Hossona et al. [28] demonstrated that tadalafil significantly improved stone expulsion compared to placebo. Additionally, various studies and meta-analyses have indicated that combining tamsulosin with tadalafil results in a significantly higher SER compared to tamsulosin alone. However, findings have been inconsistent, possibly due to differences in patient populations, study designs, or intervention durations [29].

Some studies have explored combination therapy. Chishti et al. demonstrated that combining tamsulosin with tolterodine significantly shortened the time to stone expulsion compared to tamsulosin alone [30]. The study concluded that combination therapy resulted in a significantly shorter average stone expulsion time compared to tamsulosin monotherapy. These collective findings support the concept that pharmacologic enhancement of ureteral smooth muscle relaxation can improve stone passage.

Although baseline differences were present between groups in terms of stone density and degree of hydronephrosis, which could influence spontaneous stone passage, we addressed this by conducting a multivariate logistic regression analysis. After adjusting for these confounders, L-arginine remained a statistically significant independent predictor of stone expulsion. These findings suggest that L-arginine’s effect is not solely due to favorable baseline characteristics but may reflect a true therapeutic benefit as a MET for lower ureteric stones.

The significant association between stone density and stone expulsion rate observed in our study is likely due to the physical composition and hardness of the stones. Higher-density stones may reduce their likelihood of spontaneous passage due to increased resistance to fragmentation and decreased mobility through the ureter. In contrast, lower-density stones are generally softer and more likely to pass spontaneously. These findings align with prior studies indicating that lower stone density is associated with higher rates of spontaneous passage and greater efficacy of MET [31].

This study is among the first to evaluate the use of L-arginine, a nitric oxide precursor, as a stand-alone agent for MET in patients with distal ureteral stones. While previous studies have explored alpha-blockers and PDE5 inhibitors, few have investigated agents that directly enhance NO-mediated smooth muscle relaxation. Our findings demonstrate a significantly higher stone expulsion rate with L-arginine compared to tamsulosin and placebo, suggesting a potentially more effective and well-tolerated therapeutic option. These results align with preclinical studies showing the role of NO in ureteral smooth muscle relaxation but go further by providing clinical evidence of efficacy. This positions NO-targeted therapies as a promising direction for future research in MET.

While reductions in pain episodes and analgesic use may suggest improved quality of life, this study did not use a validated QoL instrument. Future research should incorporate standardized tools to more accurately assess patient-reported outcomes.

This study has some limitations. It was conducted at a single center, which may limit generalizability. It was non-blind, introducing a potential risk of bias; no biological markers (serum NO levels) were measured, which could have provided mechanistic insight and validated the physiological effect of L-Arginine. Finally, the sample size, while sufficient to detect statistical differences, may not fully capture rare adverse events or subtle subgroup effects.

## Conclusion

L-arginine is more effective than Tamsulosin in increasing stone expulsion rate and reducing stone expulsion time, with better pain control, making it a safe and effective single medical expulsive therapy for ureteral stone.

## Data Availability

No datasets were generated or analysed during the current study.

## Appendix

CONSORT Flow Diagram
