# Correction: L-arginine role for stone lower ureter: A randomized controlled trial

**DOI:** 10.1007/s00240-026-02046-6

**Published:** 2026-09-08

**Authors:** Ahmed Mahmoud Mohammed Ahmed Elsherief, Ahmed Mahmou Riyad, Emad Abdellah Ali, Amr Alam-Eldin Ahmed, Hassan Ali Gad, Ahmed Mamdouh Abd elhameid

**Affiliations:** 1 Sohag University, Sohag, Egypt; 2 Aswan University, Aswān, Egypt

**Correction to Urolithiasis (2025) 53:109**

https://doi.org/10.1007/s00240-025-01768-3

Following publication of the original article, it was reported that the information regarding ethical approval had been inadvertently omitted from the published version.

The Ethics Statement should read as follows:

## Ethics approval and consent to participate

The study received prospective ethical approval from the Medical Research Ethics Committee (MREC), Faculty of Medicine, Sohag University, Egypt (OHRP IRB No. IRB00013006). The study protocol entitled “L-Arginine role for stone lower ureter” was reviewed and approved by the committee on 9 May 2023, prior to the commencement of patient recruitment. Written informed consent was obtained from all participants, and the study was conducted in accordance with the principles of the Declaration of Helsinki.

